# The Role of Stroma in Cholangiocarcinoma: The Intriguing Interplay between Fibroblastic Component, Immune Cell Subsets and Tumor Epithelium

**DOI:** 10.3390/ijms19102885

**Published:** 2018-09-22

**Authors:** Alessandra Gentilini, Mirella Pastore, Fabio Marra, Chiara Raggi

**Affiliations:** 1 Department of Experimental and Clinical Medicine, University of Florence, 50141 Florence, Italy; alegen1966@gmail.com (A.G.); mirella.pastore@unifi.it (M.P.); fabio.marra@unifi.it (F.M.); 2 Center for Autoimmune Liver Diseases, Humanitas Clinical and Research Center, 20089 Rozzano, Italy

**Keywords:** cholangiocarcinoma, desmoplastic stroma, tumor microenvironment, tumor-associated macrophages, cancer associated fibroblasts

## Abstract

Cholangiocarcinoma (CCA) is a severe and mostly intractable adenocarcinoma of biliary epithelial cells. A typical feature of CCA is its highly desmoplastic microenvironment containing fibrogenic connective tissue and an abundance of immune cells (T lymphocytes, Natural Killer (NK) cells, and macrophages) infiltrating tumor epithelium. This strong desmoplasia is orchestrated by various soluble factors and signals, suggesting a critical role in shaping a tumor growth-permissive microenvironment that is responsible for CCA poor clinical outcome. Indeed stroma not only provides an abundance of factors that facilitate CCA initiation, growth and progression, but also a prejudicial impact on therapeutic outcome. This review will give an overview of tumor-stroma signaling in a microenvironment critically regulating CCA development and progression. Identification of CCA secreted factors by both the fibroblast component and immune cell subsets might provide ample opportunities for pharmacological targeting of this type of cancer.

## 1. Introduction

Cholangiocarcinoma (CCA) is a severe adenocarcinoma of biliary epithelial cells [1] and along with hepatocellular carcinoma (HCC) represents a major primary liver cancer. According to its anatomical location, CCA is classified in intrahepatic (iCCA), perihilar (pCCA), and distal (dCCA) subsets [2]. Although highest CCA incidence rates have been reported in Asia (probably due to chronic infection with liver flukes), CCA incidence in Europe and North America has recently increased, and the annual mortality rates for iCCA were 9% higher in Europe during last decade. Overall, many studies show that iCCA incidence and mortality are increasing, while extrahepatic CCA (eCCA) is declining worldwide. Incidence peak occurs in the seventh decade of life with a slight male predominance [3].

Because of its silent and nonspecific clinical features, CCA is difficult to diagnose, and, in most cases, the symptoms occur when the tumor has reached an advanced stage [4]. To date, no specific markers have been identified for CCA, and the only available therapy is the surgical tumor resection that is possibly associated with chemotherapy [5]. Moreover survival rates are very low, and only 5% of patients survive up to five years from diagnosis of the tumor [6].

Similar to the pancreatic cancer, CCA often develops in nonfibrotic livers, but subsequently generates a strong desmoplastic reaction [1]. This dense stroma includes fibroblasts, immune cells, and extracellular matrix (ECM), thus creating a complex tumor microenvironment (TME). Each stroma component has a distinct role in promoting cancer development, invasion, and metastasis, as well as in conferring CCA chemo-resistance. This review will highlight the recent progresses in understanding CCA-stroma interactions that are responsible for CCA progression and drug unresponsiveness. Studies on CCA-TME are likely to contribute to new therapeutic and diagnostic strategies for this disease.

## 2. Colangio-Carcinogenesis and Inflammation

The term “colangiocarcinogenesis” refers to the complex mechanisms that lead to the malignant transformation of cholangiocytes [7]. The molecular mechanisms underlying the development of cholangiocarcinoma are not yet fully understood [8]. Persistent biliary inflammation resulting from cholestatic injury, such as primary sclerosing cholangitis (PSC), liver flukes, or infection with hepatotropic viruses appears to be the most common pre-disposing conditions for CCA.

In the conditions of bile duct damage, the inflammatory process results in release of several factors that progressively alter the biliary epithelium, culminating in upregulation of cholangiocytes growth and inhibition of cell death, and lead to inflammation, fibrosis, and cirrhosis [9]. In a chronically inflamed environment, cholangiocytes are constantly subjected to proliferative stimuli by chemokines and cytokines, growth factors, and other soluble mediators that are responsible for initiating and perpetuating the tumorigenesis [7,8,10,11,12].

Similar to PSC, infection with liver flukes is accompanied by cholangitis, fibrosis-induced obstruction of ducts, and immune responses to the flukes, as well as secondary bacterial infections that may further exacerbate inflammation and fibrosis [13]. However, when compared to the 25% lifetime risk of CCA development in PSC patients, infection with *Clonorchis sinensis* (four to six times increased risk) [13] or *Opisthorchis viverrini* (approximately fivefold increased risk [14] results in a lower relative risk of developing CCA. Inflammation appears to be to an important risk factor in this setting, because high levels of IL6 (>82.7 pg/mL) confer a >100-fold risk for CCA development in patients with *Opisthorchis viverrini* infection [15]. Taken together, these data strongly suggest a key role of microenvironment in the development of CCA-related biliary inflammation.

## 3. Microenvironment in CCA

Once biliary neoplasm develops, the presence of a well-grown fibrous stroma negatively correlates with CCA survival, and more recent studies have established its importance in maintaining tumorigenic identity of hepatobiliary tumors [16,17]. The highly desmoplastic TME represents a complex ecosystem of various cellular and non-cellular elements that are necessary for homeostasis maintenance, structural support, as well as activation of multiple signaling cascades [18,19]. Besides CCA cells, other cellular elements are mainly represented by cancer-associated fibroblasts (CAFs) and immune cell subsets (Table 1). By releasing a wide spectrum of chemokines and growth factors, these cells stimulate cancer growth, invasion, and recruitment of macrophages and T lymphocytes.

## 4. Immune Subsets in CCA Environment

The desmoplastic microenvironment in CCA presents a complex immunological landscape. Understanding the intriguing interplay between tumor cells and infiltrating immune cells may open new opportunities for therapeutic possibilities. Similar to what has been observed for CAFs, a pivotal role of tumor-associated macrophages (TAMs) in CCA progression has been convincingly validated by independent clinical studies, showing that high macrophage density predicts poor prognosis in CCA patients [20,21,22]. Moreover, the composition of CCA-infiltrating immune cells is predictive of patient survival [23,24]. Depletion of CD4^+^, CD8^+^, and Foxp3^+^ T lymphocytes, as well as the enrichment of CD163^+^ TAMs are correlated with poor prognosis of extrahepatic CCA patients, reducing recurrence free survival and inducing gemcitabine resistance after disease recurrence [24].

## 5. Tumor-Associated Macrophages

**Figure 1:**
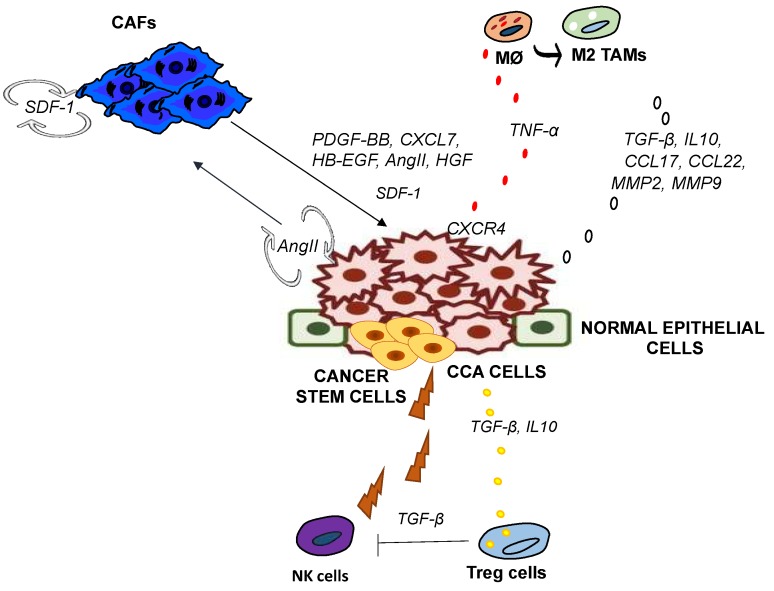
Cross-talk among CCA cells, CAFs, and tumor-associated macrophages (TAMs). Angiotensin II (AngII) released by cancer cells acts both in an autocrine and paracrine fashion. Stromal-derived factor I (SDF-1) expressed by CAFs increases upon AngII stimulation. SDF-1, AngII, PDGF-BB, HB-EGF, CXCL7, HGF released by CAFs promote tumor growth. Tumor Necrosis Factor (TNF-α (produced by TAMs enhance CXCR4 expression (receptor of SDF-1) in CCA. M2 TAMs favour tumor progression through the release of anti-inflammatory cytokines, chemokine and metalloproteinase. Treg cells mediate CCA tolerance producing anti-inflammatory cytokines and inhibiting Natural Killer (NK) antitumor activity.

Among CCA-infiltrating immune cells, TAMs play a crucial role in cancer-related inflammation by promoting tumor cell proliferation, angiogenesis, matrix turnover and suppression of adaptive immunity [25]. TAMs drive CCA onset by producing various soluble mediators, including reactive nitrogen intermediates, cytokines, including IL4, IL6, IL10, chemokines, such as CCL17, CCL18, and metalloproteinases, in particular, MMP9. In CCA, TAMs represent the main source of MMP9 [26]. Likewise, other crucial ECM remodeling-related genes, specifically MMP2, ADAM10, and ADAM17, are mainly expressed by cancer stem cell -associated TAMs [27] (Figure 1).

According to their effects on tumor, macrophages can be schematically divided into two phenotypes, M1 (classically activated macrophages) and M2 (alternatively activated macrophages). M1 macrophages are activated by IFN-γ and microbial stimuli, such as LPS, and are characterized by production of high levels of IL12, IL23 [28], and reactive oxygen intermediate and possess the capacity of antigen-presenting cells [28]. On the other hand, distinct types of M2 macrophages differentiate when monocytes are stimulated with IL4 and IL13 (M2a), with immune complexes/TLR ligands (M2b), or with IL10 and glucocorticoids (M2c). Hallmarks of M2 macrophages include the production of anti-inflammatory cytokines, such as IL10, and chemokine secretion (e.g., CCL17 and CCL22). M1 polarization promotes inflammation and suppresses tumor progression by producing pro-inflammatory and anti-tumor cytokines. In contrast, the M2 phenotype supports inflammation resolution, thus resulting in tumor progression [29,30].

TAMs represent a particular subtype of M2 macrophages and derive mainly from circulating monocytes (CD14^+^/CD16^+^), rather than from resident macrophages, which in the liver are traditionally called “Kupffer cells”. In the context of chronic injury the intrahepatic macrophages are massively expanded, following the influx of circulating monocytes more than the increase of Kupffer cells [31]. Monocyte recruitment into the liver is promoted by chemoattractant molecules, including monocyte chemoattractant protein (CCL2), colony stimulating factor (CSF)1, and vascular endothelial growth factor VEGF [32,33,34]. Intrahepatic macrophages represent an important source of CCL2 that induces the emigration of monocytes from the bone marrow [35]. Notably both receptors for CCL2 (CCR2) and for CSF1 (CSF1R1) represent an interesting therapy target, as shown by their pharmacological inhibition in clinical (PF-04136309) [36] and pre-clinical setting (PLX6134/GW2580) [37].

CD32^+^/CD68^+^ Kupffer cells (but not recruited/infiltrating CD11b^+^ macrophages) express the MER receptor tyrosine kinase (MerTK), involved in efferocytosis and CD64 (FcRI), which could be useful to distinguish CD68^+^ Kupffer cells from resident macrophages [38]. Notably, iCCA infiltrating macrophages express similar functional characteristics to the M2 subtype. Moreover, the increased density of the macrophages in iCCA is associated with a poor prognosis [39]. Recently, it has been described that periostin, a disulfide-linked cell adhesion protein favoring tumor progression [40], is secreted by CD44^+^ iCCA stem cells, and may act as a chemoattractant for M2 TAMs [41]. Furthermore, Atanasov et al. have shown that high abundance of TAMs in the tumor invasive front is associated with improved patient outcome [42]. In contrast, a high TAMs density in the tumor central area determines a strong tendency towards reduced survival. A possible explanation for these findings is that macrophage function may be altered by hypoxia and formation of necrosis in tumor central area. Both of these conditions promote M2 polarization and therefore tumor progression. Moreover, the presence of CD68^+^ TAMs in the tumor invasive front has been demonstrated to correlate with reduced tumor recurrence and to serve and an independent prognostic factor for survival [42]. It is important to note that tumor stem-like associated-TAMs displayed mixed M1-M2 molecular features, including high invasion and adhesion capability in response to a stem-like secretome that is characterized by the production of IL13, osteoactivin, and IL34, thus reinforcing the concept of TAM plasticity [27]. 

## 6. Tumor-Infiltrating Lymphocytes

Among immune cells, cytotoxic T lymphocytes recognize and eliminate tumor cells. Furthermore, due to their high plasticity, they undergo changes within tumor microenvironment, following the release of CCL2 that is produced by tumor cells, TAMs, and CAFs. Thus, tumor-infiltrating T cells acquire CD4/CD25 expression and they become T leukocyte immunosuppressive regulators (Treg) [43]. Within tumors, Tregs produce transforming growth factor-beta (TGF-β) and IL10, which contributes to an immunosuppressive environment through the inhibition of cytotoxic T cells and natural killer cells. Tregs also bind to IL2, making this cytokine unavailable in the TME and thus preventing the activation of additional immune cells [44].

In biliary tract cancers it has been observed that the deregulation of immunomodulatory transcripts in peritumoral areas may create an immunosuppressive milieu that facilitates tumor recurrence, possibly through the activation of the cytotoxic T lymphocyte antigen-4 (CTLA4) axis. CTLA4 is expressed on the surface of Tregs and requires binding to CD80 on antigen presenting cells in order to mediate the inhibitory effects on cytotoxic cells [45] (Figure 1).

## 7. Natural Killer (NK) Cells

Natural Killer cells have antitumor activity and they are characterized by CD56 or CD16 expression and the absence of CD3 expression [46]. These cells have been proposed as an innovative immune-therapy against various types of cancers lacking class I major histocompatibility complex (MHC), while in physiologic conditions the killer cell immunoglobulin-like receptor expressed by NK cells recognizes MHC I molecules that are expressed by normal cells, inhibiting the cytotoxicity of NK cells [47]. The NK pathway is mediated by NKG2D (natural-killer group 2D) [48], a lectin-like activating receptor. In cancer cells, MHC class I expression is lost or down-regulated, NK inhibitory signal is removed, and NK cells are activated to kill the malignant target cancer cells. 

Morisaki et al. have shown that combination of cytokine-activated killer (CAK) cells with cetuximab treatment prompted an increase in the cytotoxic effects on CCA cells [49]. Furthermore, it was recently shown that NK cells exert cytolytic activity against CCA, showing beneficial effects of NK cell therapy on the quality of life. In this study, the infusions of ex vivo-expanded human NK cells (SMT01) in HuCCT-1 tumor-bearing nude mice resulted in the significant inhibition of CCA growth [50] (Figure 1).

## 8. Fibroblastic Component in CCA

Constantly activated fibroblasts, identified by the expression of α-smooth muscle actin (α-SMA), are highly represented in CCA microenvironment [51]. A variety of soluble mediators produced by both neoplastic and non-neoplastic cells populating the tumor microenvironment are responsible for CAFs persistent activation [52,53]. These molecules include cytokines, chemokines, and growth factor, in particular, CCL2, CXCL12 or Stromal derived factor-1 (SDF-1), CXCL14, platelet-derived growth factor (PDGF), TGF-β, fibroblast growth factor (FGF), hepatocyte growth factor (HGF), granulocyte-macrophage colony stimulating factor (GM-CSF), and insulin-like growth factor (IGF-1) [52,53]. Several in vivo and in vitro studies highlighted the central role of α-SMA^+^ CAFs in CCA promotion and drug resistance [54], and clinical studies indicated a correlation between high CCA CAF numbers with poor patient survival [55].

Similar to what observed in HCC, a poor clinical outcome of CCA has been associated with molecular alterations in the highly desmoplastic stroma [56]. Indeed, gene overexpression of TGF-β2, laminin γ2 subunit, and osteopontin has been significantly correlated with overall survival of patients with CCA [57]. Moreover, a study conducted by Andersen et al. in 104 surgically resected CCAs, revealed that the most malignant tumor phenotype was associated with the overexpression of proinflammatory molecules in the CCA stroma, including IL6 and CXCR4 [58]. Taken together, these studies suggest an important contribution of CAFs in CCA development [56].

Although CAFs are one of the most abundant stromal cell types in the CCA TME [53], potential sources of CAFs in CCA remains unclear, and CAFs may potentially derive from quiescent hepatic stellate cells (HSCs), portal fibroblasts, bone marrow-derived fibroblasts, as well as cholangiocytes-derived fibroblasts via epithelial mesenchymal transition. HSCs are a major source of myofibroblasts in multiple models of biliary liver fibrosis [59], and it is likely that, among stromal cells, these cells represent the main precursors of CAF [56]. However, the formal fate mapping studies are still lacking.

## 9. Impact of CAFs on CCA Cells

The first study indicating a role of myofibroblasts in CCA development was conducted by Okabe et al. [60], who observed an increase in cancer cell invasion and growth, when the intrahepatic CCA cell lines were co-cultured with LX2. Moreover, by analyzing 46 human iCCA specimens, a positive correlation between α-SMA expression and poorer outcome of the patients was found. Since α-SMA^+^ cells that were observed in iCCA specimens also express desmin or glial fibrillary acidic protein (GFAP), it is conceivable that they are derived from HSCs. Additional in vitro studies from the same group were conducted in two HSCs cell lines cocultured with iCCA conditioned medium (CM), and viceversa [61]. Increased HSCs activation and proliferation in presence of iCCA CM was observed, and the exposure of CCA cells to HSC CM resulted in increased survival and invasiveness. Moreover, CM obtained by iCCA-activated HSCs, induced tube formation in HUVEC, suggesting that tumor cells can stimulate CAFs to produce angiogenic factors. Notably, in the early stages of CCA carcinogenesis, α-SMA^+^ CAFs induce the proliferation of non-neoplastic biliary epithelial by inducing apoptosis inhibition [55].

## 10. CCA Fibroblasts and Secreted Factors

CAFs can fuel CCA overgrowth via paracrine mechanisms [53], involving secretion of several factors (Table 2), such as HGF, TGF-β, PDGF-BB, heparin-binding epidermal growth factor (HB-EGF), and SDF-1 (Figure 1) [51].

CXCL12 belongs to the chemokine family, which, together with interleukins, regulates the trafficking of leukocytes and endothelial cells to sites of inflammation, infection, and malignancy. The CXCL12/CXCR4 axis is implicated in the invasion and migration of cancer cells, including CCA cells [62,63]. Okamoto et al. [64] showed that CXCR4 is expressed in both CCA cells and CAFs in vivo and in vitro, while CXCL12 is mainly expressed by CAFs in vivo and HSCs in vitro*.* Moreover CXCL12 released by stellate cells induced an increase in survival and activation of HSCs, as well as the enhancement of iCCA migration, acting therefore both in an autocrine and a paracrine fashion. Actually, in a study conducted by our group, a strong CXCR4 expression was observed in all CCA tissues examined, whereas CXCL12 was mainly released by primary human HSCs and not by iCCA cells [65]. In iCCA cells, we observed an increase of migration induced by SDF-1/CXCR4 dependent on ERK1/2 and AKT activation. We also detected an increase of SDF-1/CXCR4-induced survival in the same cells, dependent on a reduced activation of PARP [65].

PDGF is another growth factor that is secreted by CAFs and it plays an important role in mediating cross talk between cholangiocytes and fibroblasts in animal models of biliary duct inflammation and fibrosis [66,67]. Among the five isoforms of PDGF (AA, BB, AB, CC, and DD), CAFs/HSCs express mainly PDGF-BB [68,69,70], while one of its receptor, PDGFR-β, is expressed in CCA cells [71]. In a recent study, HSCs decreased TRAIL-induced apoptosis of CCA cell lines via release of PDGF-BB, and cytoprotection that is induced by PDGF-BB was dependent on Hedgehog (Hh) signaling [72]. Indeed, PDGF-BB caused translocation of smoothened (SMO) (a transducer of Hh signaling) to the plasma membrane of CCA cell lines, a PKA-dependent process. Finally, in an orthotopic rat CCA model, the presence of PDGF-BB in myofibroblasts and PDGFR-β in CCA cells were confirmed, and after cyclopamine administration (a SMO inhibitor), tumor apoptosis increased and tumor size and weight decreased. Of note, imatinib mesylate, which is an inhibitor of PDGFR-β, retained the same effects as cyclopamine in the rat CCA model [73]. Further studies on Hh signaling revealed a role of this signaling pathway in the proliferation, migration, and invasion of CCA cells as well as in angiogenesis development in co-implant (CCA cells and HSCs) xenograft models in vivo*.* Notably, cyclopamine treatment decreased tumor growth and microvessel density only in co-implant and not in single implant xenograft group, suggesting an activation of Hh signaling in a paracrine manner that is likely mediated by HSCs [74]. Moreover, also CAFs overexpress PDGFR-β, which promotes their own cell proliferation and survival [75].

Notably, therapeutic targeting of the PDGFR-β (imatinib mesylate) [76] might interfere with the interplay of CAFs-CCA cells, whereas small molecule pro-apoptotic compounds, so called BH-3 mimetic, inhibit the BCL-2 protein [54], inducing apoptosis selectively depleting CAFs. Accordingly, BH3 mimetics reduced tumor growth and metastasis and improved survival in a murine model of CCA [54,77]. Similarly, targeting CAFs with a TGF-β antagonist reduced both fibrosis and CCA development in thioacetamide-treated rats [78].

Modulatory effects of CAFs on CCA may also be dependent on the over-expression of epidermal growth factor receptor (EGFR). Mutations, activation, and overexpression of EGFR are associated with a poor outcome in CCA patients [58,79,80]. Among EGFR ligands, HB-EGF has been found to play a role in liver cancer development [81,82]. Clapéron et al. observed that in a xenograft model of CCA cells coinjected with primary human liver myofibroblasts, treatment with gefitinib (an inhibitor of EGFR tyrosine kinase activity) reduced tumor growth and angiogenesis as compared to vehicle-treated mice [83]. Moreover, immunohistochemistry of human CCA samples revealed positivity for HB-EGF in myofibroblasts and tumor cells, while EGFR was present in CCA cells. Cultured myofibroblasts expressed HB-EGF and their CM induced migration and invasion in several CCA cell lines through the phosphorylation of EGFR and activation of its signaling. In addition TGF-β, released by CCA cell lines, induced HB-EGF production by myofibroblasts. Interestingly upon HB-EGF incubation, CCA cells enhanced TGF-β expression, suggesting a paracrine loop of these growth factors involving cancer and stroma cells.

Among key molecules involve in CCA proliferation [84], angiotensinII (AngII) and its receptor AT-1 may have a role in biliary tumorigenesis [85]. Beside elevated expression of AngII in CCA tissues as well as high AT-I receptor immunoreactivity in both epithelial and stromal cells, AngII induced survival of iCCA cells as well as an increase in HSC activation (LI90), indicating a role for the AngII-AT-1 axis as a regulator of tumor progression, both in an autocrine and paracrine fashion. CXCL7 is an additional chemokine which plays a role in tumor growth in human cancers [86,87]. CXCL7 positive expression was found in 66% of CCA specimens, while only 23% of non-tumor tissue showed immunoreactivity for this chemokine. Moreover CXCL7 expression was associated with advanced progression and poorer prognosis of CCA patients. In vitro data showed that CXCL7 promotes CCA cell proliferation and invasion, through the activation of AKT signaling, both in autocrine and paracrine manners, as mediated by HSCs.

CXCL15 is another chemokine released by CCA, and induced invasion and migration of these cells in an autocrine mode through activation of ERK 1/2 and AKT. Notably, IL1β, released by hepatic stellate line cells, could enhance CXCL15 expression by paracrine signaling. In addition, epithelial expression of CXCL15 in CCA tissues correlated with α-SMA expression, recruitment of tumor-infiltrating neutrophils and poor prognosis after hepatic resection [88]. 

Some of the above-listed soluble mediators molecules are currently investigated in clinical and preclinical studies (Table 3).

## 11. Fibroblasts in C*lonorchis sinensis* Derived CCA

Infection by *Clonorchis sinensis* is one of the most diffused parasitic diseases in East Asia. This infection can lead to hepatobiliary damage, inflammation, hepatic fibrosis, and CCA [92]. Several studies had reported that in experimental animals *Clonorchis sinensis* could induce hepatic fibrosis, localized in the hepatic sinusoids in the early stage of the infection, suggesting that excretory/secretory products of the parasite could contribute to developing the fibrogenic process [93,94]. A lysophospholipase, clonorchis sinensis lysophospholipase (*Cs*LysoPLA), cloned and characterized by Ma et al. [95], is a possible candidate to stimulate HSCs activation and fibrosis. It was reported that it stimulates the growth of the parasite by breaking down complex lipids [96]. Indeed, this protein induced the activation of LX2 and was expressed at higher level at metacercariae stage, corresponding to the initiation of the infection [97]. Moreover recent data showed that the activation of LX2, induced by *Cs*LysoPLA, is mediated by JNK signaling and that BALB/c mice inoculated abdominally with recombinant *Cs*LysoPLA, accumulated collagen and increased p-JNK expression in the liver [98]. *Cs*LysoPLA could also stimulate macrophages (RAW264.7) to produce IL25 that, in turn, induced stimulation of LX2 [99]. However, opposite results on the role of *Cs*LysoPLA in fibrosis were reported in a study conducted by Zhou L. et al. showing that LX2, stimulated with *Cs*LysoPLA, attenuated HSC- induced activation by TGF-β1 through the decreased the phosphorylation of Smad3, JNK2, and ERK1/2 [100].

Other components of excretory/secretory products of the parasite have been shown to play a role in liver fibrogenesis. Fructose-1,6-bisphosphatase from *Clonorchis sinensis* was detected in the bile duct epithelial cells in proximity to worms and recombinant fructose-1,6-bisphosphatase could bind to membrane of LX2 and stimulate activation [101]. Ferritin heavy chain produced by *Clonorchis sinensis* is involved in liver inflammation. It causes free-radical production and increased levels of the pro-inflammatory mediators IL1β and IL6 in LX2 [102]. Biliary fibrosis induced by this parasite also involves the activation of toll-like receptor 4 (TLR4), highly expressed in activated HSCs during *Clonorchis sinensis* induced fibrosis [103]. In TLR4 mut mice infected by the parasite, the deposition of collagen and the activation of HSCs were significantly lower than in wild type mice [104]. These data suggest an important role of HSCs in liver flukes infection and fibrosis.

## 12. Cross-Talk between CCA Fibroblasts and Immune Cells

The permanent activation of CAFs makes these cells capable of recruiting immune cells from the bloodstream to tumor site through several growth factors, together with other cell types, in particular VEGF, FGF, cytokines, and chemokines, such as CCL2, CXCL12, and CXCL14 [53]. These complex cellular mechanisms favor the establishment of a pro-fibrotic and pro-angiogenic environment, which contributes significantly to the initiation and progression of CCA [105]. Recent reports suggest that both CAFs and TAMs promote CXCR4 expression in CCA cells through the production of tumor necrosis factor (TNF)-α and hepatocyte growth factor (HGF), respectively [105,106] highlighting a complex network between epithelial and stromal cells. Additionally, it has been shown that the interaction of CAFs with CCA cells promote IL1β and CXCL5 production, in CAFs and CCA cells, respectively [88]. Cancer cell-derived CXCL5 acts as an immune cells chemoattractant [88]. CAF-derived thrombospondin-1 exerts immunosuppressive effects via TGF-β activation and direct interaction with immune cells [107,108]. Meanwhile, Matrix Metalloproteinases (MMPs) are chemotactic for leukocytes and modulate their proliferation as well as cytokine release [109,110]. TGF-β is a potent suppressor of antitumor immunity via effects on NKs, dendritic cells, macrophages, neutrophils, CD8^+^ and CD4^+^ effector cells, and Tregs [111] suggesting a contribution of CAFs to immunosuppression. Cross-talk among CAFs and TAMs are not entirely understood and further studies are necessary to identify CAF effects on the recruitment as wells function of CCA-TAMs.

## 13. Concluding Remarks

CCA desmoplastic environment co-evolves together with tumor mass supporting neoplastic growth, as well as restricting drug delivery. A multitude of signals are released, facilitating close interactions between the stromal and epithelial compartments, thus providing new druggable targets for treatment of desmoplastic tumors, including CCA [112].

Among several immunotherapeutic approaches for CCA treatment, strategies that are based on immune checkpoint blockade have produced positive results and paved the way for their use in clinical setting. In order to evade immune surveillance as mechanism of resistance, cancer cells frequently manipulate immune checkpoints. Blockade of immune checkpoints reconstitutes normal anti-tumor immunity and this represents a new CCA therapeutic approach. Monoclonal antibodies to the CTLA-4 (a protein regulating T cell tolerance) have entered clinical cancer therapy and have been proven highly effective in several types of tumor. Likewise, programmed death 1 (PD-1) and programmed death ligand 1 (PD-L1) have been described to be upregulated in a specific set of CCA. Thus, different molecules are under investigation in clinic: anti-PD-1 antibody (pembrolizumab) in phase 1/2 studies show promising efficacy in CCA, with about 40% response rate and the PD-L1 inhibitor (nivolumab) has just been approved for HCC, but not yet for CCA [113,114]. Along with immunotherapy, interfering with the CCA-CAF crosstalk may also represent a valid therapeutic alternative, as indicated by the inhibition of the PDGFR-β (imatinib mesylate). 

Therefore, these encouraging results suggest that targeting cancer associated fibroblasts and/or reversing immune checkpoint may have a potential efficacy in CCA treatment. Therapies targeting desmoplastic microenvironment may be effective in improving patient response to conventional chemotherapy, thus suggesting a potential effectiveness of CCA combinatorial therapies.

## Figures and Tables

**Table 1:** Cellular components of tumor stroma.

Lineage	Role in Cholangiocarcinoma
TAMs	Promotion of CCA proliferation, angiogenesis and tumor remodeling; Immunosuppressive action; Production of interleukins and chemokines; Secretion of matrix metalloproteinases (MMPs): MMP2 and MMP9
Treg cells	Immunosuppressive action; Secretion of TGF-β and IL10 that inhibit the antitumor activity of cytotoxic T-cells and natural killer cells
NK cells	Antitumor and cytolytic activity
CAFs	Secretion of MMPs involved in tumor remodeling; Production of several growth factors, of chemokines and interleukins

**Table 2:** Effects of soluble factors secreted by Cancer Associated Fibroblasts (CAFs) on Cholangiocarcinoma (CCA) cells.

Soluble Factors	Function
CXCL12	By binding to CXCR4, expressed in CCA cells, induces an increase in migration in a ERK1/2- and AKT- dependent manner and an increase in survival through a reduced activation of PARP.
PDGF-BB	Induces an increase in CCA proliferation and survival in a Hedgehog signaling-dependent manner.
HB-EGF	Promotes migration and invasion of CCA cells through phosphorylation of EGFR and activation of its signaling.
AngII	Facilitates tumor progression by binding AT-1 receptor, expressed in CCA cells. Induces survival of CCA cells and HSCs promoting tumor growth in an autocrine and paracrine fashion.
CXCL7	Promotes CCA cells proliferation and invasion, through activation of AKT signaling.

**Table 3:** Some of CCA soluble factor-receptors as potential druggable targets.

Soluble Factors	Inhibitors of Respecitve Receptors	Pre-Clinical or Clinical Studies	References
PDGF-BB	IMATINIB MESYLATE (inhibitor of PDGFR-β)	Pre-clinical studies	[73]
	SORAFENIB(inhibitor of PDGFR-β)	Clinical studies	[89]
	CYCLOPAMINE(SMO inhibitor)	Pre-clinical studies	[71]
HB-EGF	LAPATINIB(inhibitor of EGFR)	Clinical studies	[90]
AngII	TELMISARTAN(inhibitor of AngIIR)	Preclinical studies	[91]
	LOSARTAN(inhibitor of AngIIR)	Pre-clinical studies	[91]

## Abbreviations

CCA	Cholangiocarcinoma
iCCA	Intrahepatic Cholangiocarcinoma
pCCA	Perihilar Cholangiocarcinoma
dCCA	Distal Cholangiocarcinoma
eCCA	Extrahepatic Cholangiocarcinoma
HCC	Hepatocellular Carcinoma
TME	Tumor Microenvironment
TAMs	tumor-associated macrophages
CTLA4	Cytotoxic T Lymphocyte Antigen-4
MHC	Major Histocompatibility Complex
GFAP	Glial Fibrillary Acidic Protein
CM	Conditioned Medium
CAFs	Cancer Associated Fibroblasts
HSCs	Hepatic Stellate Cells
HGF	Hepatocyte Growth Factor
AngII	AngiotensinII
EGFR	Epidermal Growth Factor Receptor
HB-EGF	Heparin-Binding Epidermal Growth Factor
TLR4	Toll-Like Receptor 4
ECM	Extracellular Matrix
MMPs	Matrix Metalloproteinases
PSC	Primary Sclerosing Cholangitis
PDGF	Platelet-Derived Growth Factor
TGF-β	Transforming Growth Factor-beta
FGF	Fibroblast Growth Factor
HGF	Hepatocyte Growth Factor
GM-CSF	Granulocyte-Macrophage Colony Stimulating Factor
IGF-1	Insulin-like Growth Factor
SDF-1	Stromal derived factor-1
Hh	Hedgehog
*Cs*LysoPLA	Clonorchis sinensis Lysophospholipase
TNF-α	Tumor Necrosis Factor
PD-1	Programmed Death 1
PD-L1	Programmed Death Ligand 1
VEGF	Vascular Endothelial Growth Factor
